# Soluble Semaphorin 4D Serum Concentrations Are Elevated in Critically Ill Patients with Liver Cirrhosis and Correlate with Aminotransferases

**DOI:** 10.3390/diagnostics14040370

**Published:** 2024-02-08

**Authors:** Samira Abu Jhaisha, Philipp Hohlstein, Eray Yagmur, Vera Köller, Maike R. Pollmanns, Jule K. Adams, Theresa H. Wirtz, Jonathan F. Brozat, Lukas Bündgens, Karim Hamesch, Ralf Weiskirchen, Frank Tacke, Christian Trautwein, Alexander Koch

**Affiliations:** 1 Department of Medicine III, RWTH-University Hospital Aachen, Pauwelsstrasse 30, 52074 Aachen, Germanykhamesch@ukaachen.de (K.H.);; 2 Institute of Laboratory Medicine, Western Palatinate Hospital, 67655 Kaiserslautern, Germany; eyagmur@westpfalz-klinikum.de; 3 Department of Hepatology and Gastroenterology, Charité—Universitätsmedizin Berlin, Campus Virchow-Klinikum (CVK) and Campus Charité Mitte (CCM), Augustenburger Platz 1, 13353 Berlin, Germany; frank.tacke@charite.de; 4 Institute of Molecular Pathobiochemistry, Experimental Gene Therapy and Clinical Chemistry (IFMPEGKC), RWTH-University Hospital Aachen, Pauwelsstraße 30, 52074 Aachen, Germany; rweiskirchen@ukaachen.de

**Keywords:** soluble Semaphorin 4D, sSema4D, sCD100, ICU, sepsis, prognosis, organ failure, liver, inflammation

## Abstract

Semaphorin 4D (Sema4D), also known as CD100, is a multifunctional transmembrane protein with immunoregulatory functions. Upon the activation of immune cells, soluble Semaphorin 4D (sSema4D) is proteolytically cleaved from the membrane by metalloproteinases. sSema4D levels are elevated in various (auto-)inflammatory diseases. Our aim was to investigate sSema4D levels in association with sepsis and critical illnesses and to evaluate sSema4D’s potential as a prognostic biomarker. We measured sSema4D levels in 192 patients upon admission to our medical intensive care unit. We found similar levels of sSema4D in 125 patients with sepsis compared to 67 non-septic patients. sSema4D levels correlated with leukocytes but not with other markers of systemic inflammation such as C-reactive protein or procalcitonin. Most interestingly, in a subgroup of patients suffering from pre-existing liver cirrhosis, we observed significantly higher levels of sSema4D. Consistently, sSema4D was also positively correlated with markers of hepatic and cholestatic injury. Our study suggests that sSema4D is not regulated in sepsis compared to other causes of critical illness. However, sSema4D seems to be associated with hepatic injury and inflammation.

## 1. Introduction

Semaphorin 4D (Sema4D), also known as CD100, is a multifunctional 150 kDa transmembrane protein expressed in various tissues, such as endothelial cells, platelets, and immune cells including T-cells, B-cells, neutrophils and monocytes. Upon cell activation, soluble Semaphorin 4D (sSema4D) or soluble CD100 (sCD100), a 120 kDa protein, is proteolytically cleaved from the membrane by metalloproteinases to form a soluble homodimer, which retains the same functions as its membrane-bound form [1,2,3,4]. As part of the semaphore family, Sema4D is a key player in axon guidance during neural development, mainly in a repulsive manner [5,6]. In recent years, Sema4D has gained attention for its role in immune response and autoimmunity and has been investigated as a prognostic biomarker and potential therapeutic target. There is currently a single-center observational study ongoing that will compare blood concentrations of Semaphorins 1 to 7 in patients with early septic shock to those of postoperative patients suffering systemic inflammatory response syndrome (clinicaltrials.gov, NCT02692118) [7].

Sema4D primarily exerts its effects through receptors of the plexin family, namely Plexin B1, B2 and C1, which are found in many tissues and cells except the immune system. These cells control axon-guidance processes and endothelial cell migration [6,8,9]. Among the plexin family, Plexin B1 is the main receptor with the highest affinity for Sema4D [10,11].

CD72 is the main receptor for Sema4D in the immune system, although it binds to Sema4D with a much lower affinity than plexins [11,12]. CD72 is expressed in lymph nodes and the spleen as well as B cells, dendritic cells and natural killer cells, regulating their activation, proliferation and migration [12,13,14,15,16,17].

During coagulation, Sema4D performs a dual role. At the beginning of thrombus formation, Sema4D binds to the receptors of nearby platelets, which promotes thrombus formation. This is followed by the shedding of Sema4D from the cell surface as the thrombus grows. This allows sSema4D to interact with other cell types such as endothelial cells and monocytes [4].

In autoimmune disease, sSema4D has been described as pro-inflammatory and was found to correlate with disease activity, making it a promising biomarker in this context. So far, it has best been characterized in patients with rheumatoid arthritis (RA), patients with antineutrophil cytoplasmic antibody (ANCA)-associated vasculitis (AAV) and children with Kawasaki disease [18,19,20].

In patients with rheumatoid arthritis, serum sSema4D levels were shown to be significantly higher than in healthy controls; however, in patients with osteoarthritis, ankylosing spondylitis and systemic lupus erythematodes, sSema4D levels were not increased compared to healthy controls. In patients with RA, serum levels of sSema4D were positively correlated with disease activity markers, C-reactive protein (CRP) levels, rheumatoid factor titers and bone metabolic markers. Yoshida et al. demonstrated a significant decrease in sSema4D levels after 6 months in patients who responded to a biological DMARD therapy. This decrease was not observed in patients who only moderately responded or did not respond at all to a 6-month course of DMARD therapy [18]. The increase in sSema4D serum levels was accompanied by a decrease in cell surface expression especially on CD3+ and CD14+ cells, suggesting that the elevation of sSema4D resulted from increased shedding from membrane-bound Sema4D, particularly by ADAMTS-4. Treatment of CD14+ monocytes with sSema4D induced the production of the proinflammatory cytokines IL-6 and TNF-α.

Patients with AAV have significantly higher serum levels of sSema4D compared to patients with bacterial infections and healthy controls. Furthermore, sSema4D levels are positively correlated with vasculitis activity scores and neutrophil counts, but no correlation with CRP or MPO-ANCA titers has been shown. In addition, Nishide et al. showed that the cell surface expression of Sema4D in polymorphonuclear cells was reduced in patients with AAV compared to healthy controls, which was attributed to the shedding of Sema4D from activated neutrophils, primarily mediated via the metalloproteinase ADAMTS-17 [19].

Wang et al. recently showed that Sema4D is highly expressed in liver samples from patients with fibrotic liver disease compared to healthy controls. The main sources of Sema4D in fibrotic liver are activated hepatic stellate cells followed by hepatocytes and endothelial cells. Using different mouse models, they demonstrated that Sema4D knockout suppresses liver fibrosis, which is partly mediated by regulating the balance of Th1, Th2, Th17 and T-bet positive Treg cells [21].

Given its complex function regarding immune response and immunological cell migration, we hypothesized that sSema4D may play an important role in the setting of critically ill and septic patients and serve as a biomarker for the prediction of disease severity or survival. So far, sSema4D has not been investigated in this setting. Therefore, we conducted a study in our tertiary care medical intensive care unit (ICU) with a cohort of septic and non-septic patients to elucidate sSema4D’s clinical relevance and potential prognostic value.

## 2. Materials and Methods

### 2.1. Selection and Inclusion of Patients

In this observational cohort study, we enrolled 192 patients, in a prospective manner, who were consecutively admitted to our tertiary care medical intensive care unit at the Department of Gastroenterology, Digestive Diseases, and Intensive Care Medicine of University Hospital RWTH Aachen, Germany, from 2006 to 2012. Patients above 18 years of age and with written informed consent for inclusion in the study (from the patient, their spouse or appointed legal guardian) were considered for inclusion. Patients with an expected short-term ICU stay (<48 h), patients admitted from another ICU and patients with poisoning were excluded, as previously described [22,23,24]. The Third International Consensus Definitions for Sepsis and Septic Shock (Sepsis-3) were used to differentiate between septic and non-septic patients, and treatment was conducted according to guidelines [25]. Our study was approved by our local ethics committee and conducted in accordance with the ethical standards stated in the Declaration of Helsinki (reference number EK150/06). For the assessment of long-term survival, the patient, their spouse or primary care physician were contacted at 6-month intervals for 2 years after discharge from the ICU.

### 2.2. Laboratory Analysis of Circulating Soluble Semaphorin4D in Patient Serum

Blood samples were collected from patients upon admission to the ICU. Following centrifugation for 10 min at 4 °C, serum was aliquoted and stored at −80 °C until further analysis. The quantitative sandwich enzyme-linked immunosorbent assay (ELISA) for soluble Semaphorin 4D (Soluble Semaphorin 4D ELISA kit, BI-20405, Biomedica Medizinprodukte GmbH, Vienna, Austria) was performed according to the manufacturer’s instructions [26]. Measurements of sSema4D were performed without knowledge of any clinical or laboratory data from the patients.

### 2.3. Statistical Analysis

Statistical analyses were performed and graphs were created using SPSS version 29 (SPSS, Chicago, IL, USA) and the packages NumPy version 1.21.5 [27], Pandas version 1.4.4 [28], Matplotlib version 3.5.2 [29], Seaborn version 0.11.2 [30], Pingouin version 0.5.3 [31], Scikit-learn version 1.0.2 [32] and Lifelines [33] in Jupyter Notebooks version 6.5.4 [34] using Python version 3.11 [35]. Due to the skewed distribution of most parameters, data are presented as the median and range. As a normal distribution could not be assumed, either the two-tailed Mann–Whitney U test or the Chi-squared test was applied to assess differences between groups. In cases with more than two groups, the Kruskal–Wallis test was applied. Statistical significance was assumed where *p* < 0.05 for all calculations. Spearman’s rank correlation test was used to analyze the associations between sSema4D and the other variables. The Youden Index (the sum of sensitivity and specificity minus one) was calculated and then used as the optimal cut-off value for the prognosis analyses. A receiver operating characteristic (ROC) curve with the corresponding area under the curve (AUC) was determined to evaluate the quality of the prognostic marker. Kaplan–Meier curve analysis, including 95% confidence intervals, was applied to examine differences in survival. A log-rank test was used to assess significance. 

## 3. Results

### 3.1. Soluble Semaphorin 4D Serum Concentrations Do Not Differ between Septic and Non-Septic Critically Ill Patients

A total of 192 patients were included in this study. Of these, 125 were admitted to the ICU due to sepsis and 67 were admitted due to other critical illness. The median age of the study cohort was 64.5 years, ranging from 18 to 89, and there was no significant difference between septic and non-septic patients. Of these patients, 113 (58.9%) were male and 79 (41.1%) were female. There were no differences in age, gender distribution, comorbidities (measured using the Charlson Comorbidity Index) or mortality between the two groups.

**Figure 1:**
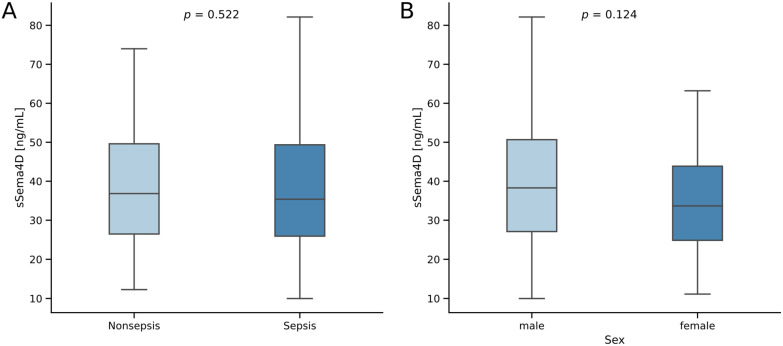
Serum sSema4D concentrations in critically ill patients with and without sepsis (**A**) and comparison between the sexes (**B**). Sample sizes: patients *n* = 192, non-sepsis *n* = 67, sepsis *n* = 125. Significance of the difference between groups was assessed using the Mann-Whitney U test. *p*-values < 0.05 were considered statistically significant.

We did not observe any difference in sSema4D concentrations between septic and non-septic critically ill patients (Figure 1).

As expected, septic patients more frequently required mechanical ventilation (73.6% vs. 57.5%, *p* = 0.036) as well as vasopressor therapy (70% vs. 47.4%, *p* = 0.005). Disease severity, as assessed using the APACHE II score, was higher in septic patients (median of 18 vs. 16 points, *p* = 0.039) and organ failure, as assessed using the SOFA score, was more severe in sepsis patients (median of 11 vs. 7 points, *p* = 0.006). Consistent with this, septic patients required longer ICU treatment than non-septic patients (median of 10 vs. 6 days, *p* < 0.001; Table 1).

### 3.2. Soluble Semaphorin 4D Levels Are Not Associated with Disease Etiology

In our cohort, the most common type of infection in sepsis patients was pulmonary (55.2%). Other origins of sepsis were abdominal (15.2%) or urogenital (8%). The remaining sepsis patients (21.6%) were treated due to blood-stream infections, skin infections or an unknown site of infection. We observed a trend towards higher sSema4D levels in sepsis patients with other sites of infection, with a median of 44.62 ng/mL (range: 15.70–184.60 ng/mL) compared to patients with a pulmonary focus (median: 33.11 ng/mL; range 9.99–240.00 ng/mL), an abdominal focus (median: 36.69 ng/mL; range: 11.12–160.27 ng/mL) or a urogenital focus (median: 34.51 ng/mL; range 16.30–82.13 ng/mL). However, these differences were not statistically significant (*p* = 0.059) (Table 2).

The non-septic critically ill patients were admitted to our ICU due to cardiocirculatory disorders (19.4%), advanced liver disease (19.4%), respiratory failure (14.9%) and a variety of other diseases (46.3%). In this group, the regulation of sSema4D levels was not observed (*p* = 0.809) (Table 2).

### 3.3. Soluble Semaphorin 4D Serum Concentrations Are Increased in Patients with Liver Cirrhosis and Positively Correlated with Liver Function Tests

We set out to test whether certain comorbidities had an influence on sSema4D concentrations. Patients with a history of diabetes, coronary artery disease, hypertension, chronic obstructive lung disease, chronic alcohol abuse and active malignancy did not have altered sSema4D concentrations (Table 3).

However, patients with pre-existing liver cirrhosis (*n* = 20) showed significantly higher sSema4D levels, with a median of 47.25 ng/mL (range: 15.14–163.01 ng/mL, *p* = 0.02). Etiologies of liver cirrhosis were alcohol related (*n* = 8) or due to chronic hepatitis B (*n* = 2), chronic hepatitis C (*n* = 2), primary biliary cholangitis (*n* = 2), non-alcoholic steatohepatitis (*n* = 1), cardial dysfunction (*n* = 1) or cryptogenic (*n* = 4). Moreover, sSema4D was positively correlated with liver function tests. We observed a weak correlation between sSema4D and alanine aminotransferase (ALT, Spearman’s r = 0.160, *p* = 0.027) and a correlation of moderate strength with aspartate aminotransferase (AST, Spearman’s r = 0.258, *p* < 0.001) as markers of hepatic injury and inflammation. The cholestatic parameters bilirubin (Spearman’s r = 0.170, *p* = 0.019) and gamma-glutamyl transpeptidase (γGT, Spearman’s r = 0.237, *p* = 0.001) also showed weak and moderate correlations with sSema4D, respectively (Table 4).

### 3.4. Soluble Semaphorin 4D Serum Concentrations Correlate with Leukocyte Count but Not with Other Markers of Systemic Inflammation

To identify the factors that influence sSema4D levels, we performed extensive correlation analyses between sSema4D and different markers of inflammation, laboratory values, clinical scores and demographics. sSema4D correlated with leukocyte count (Spearman’s r = 0.144, *p* = 0.048) but not with other established markers of inflammation such as C-reactive protein, procalcitonin and interleukin-6 or interleukin-10. We observed a weak correlation between sSema4D and triglycerides (Spearman’s r = 0.201, *p* = 0.012) while other metabolic markers, such as cholesterol, high-density lipoprotein, low-density lipoprotein, glycosylated hemoglobin A1, blood glucose, insulin and C-peptide, showed no correlation. Body mass index, age, markers of renal function and plasmatic coagulation markers also showed no correlation. Underlining the fact that sSema4D concentrations did not differ between septic and non-septic patients, we found no correlation with clinical markers of disease severity (APACHE-II, SOFA, Horovitz, vasopressor demand and FiO_2_ demand) (Table 4).

### 3.5. Soluble Semaphorin 4D Serum Concentrations Do Not Predict Short-Term or Long-Term Mortality

To examine the potential prognostic value of sSema4D, we compared the baseline sSema4D serum levels of surviving and deceased patients at different timepoints.

**Figure 2:**
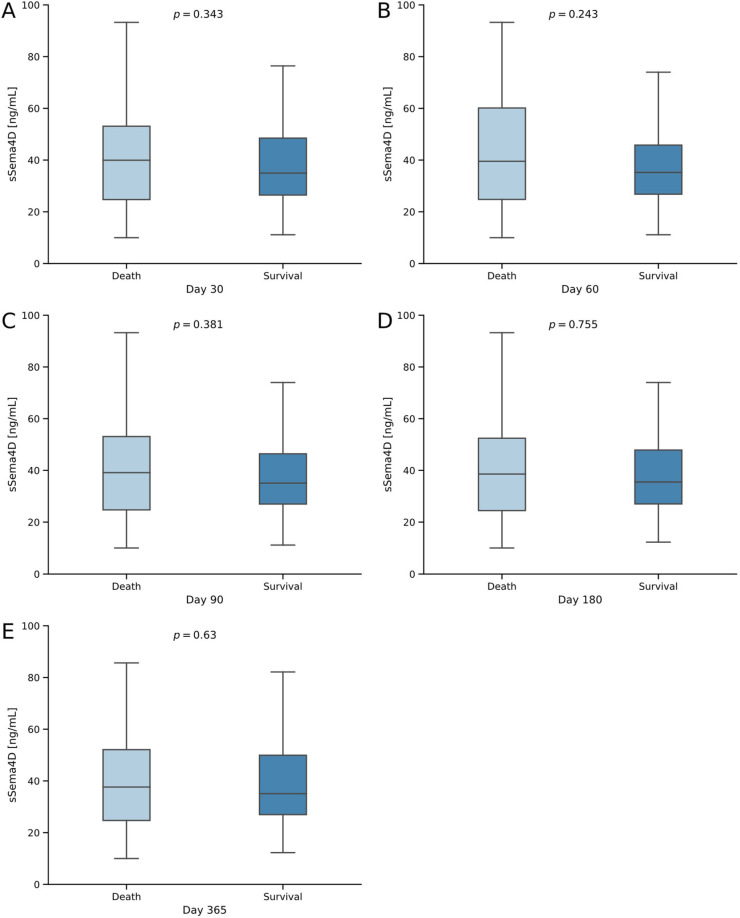
sSema4D levels in a consecutive survival analysis of critically ill patients treated in the ICU. (**A**–**E**) Survival status at days 30 through 365. The sample size was *n* = 192 patients. Significance of the difference between groups was assessed using the Mann–Whitney U test. *p*-values < 0.05 were considered statistically significant.

**Figure 3:**
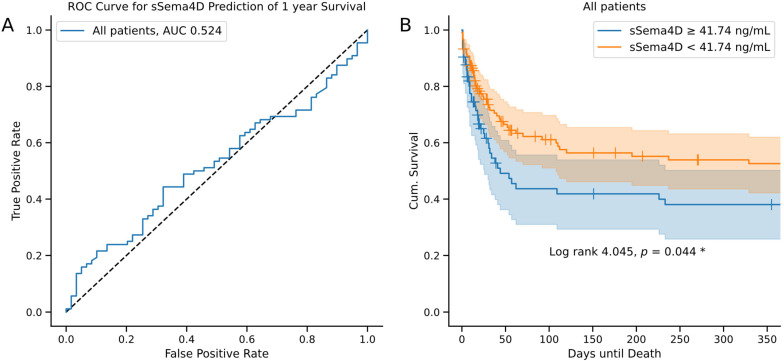
Receiver operating characteristic (ROC) curves for the prediction of one-year survival using serum sSema4D levels in all patients (**A**). Kaplan–Meier curves for sSema4D < 41.74 ng/mL (orange) and ≥41.74 ng/mL (blue) in all patients (**B**). Censored events are indicated by a crossing vertical line. Cut-off values of the Kaplan–Meier curve were determined using the Youden Index for all patients. Sample size: patients *n* = 192. Significance of the difference between groups was assessed using the log-rank test. *p*-values < 0.05 were considered statistically significant and are denoted by an asterisk (“*”). Abbreviations: AUC: area under curve. Shaded areas in the Kaplan–Meier curves represent the 95% confidence intervals.

There was no significant difference in sSema4D at the baseline between the groups at 30 days after admission (*p* = 0.343; Figure 2A), 60 days after admission (*p* = 0.243; Figure 2B), 90 days after admission (*p* = 0.381; Figure 2C), 180 days after admission (*p* = 0.755; Figure 2D) or a year after admission (*p* = 0.630; Figure 2E) to the ICU. However, at all the timepoints mentioned above, baseline median sSema4D levels were lower in surviving patients than in deceased patients. We performed a receiver operating characteristic (ROC) curve analysis in which sSema4D levels showed an area under the curve (AUC) of 0.524 for the prediction of survival after one year (Figure 3A). In addition, we conducted Kaplan–Meier curve analyses using the Youden Index to establish the ideal cut-off value for the prediction of survival at the one-year observation period for all patients. This showed a trend towards improved survival for patients with baseline sSema4D < 41.74 ng/mL (log-rank 4.045, *p* = 0.044; Figure 3B). Although the log-rank test, which compares the median survival times between groups, suggests a significant difference, the 95% confidence intervals overlap during the whole observation period of one year. Potentially, a significant difference in survival is reached after one year.

## 4. Discussion

Sema4D has gained recent attention due to its diverse functions in the immune system; it is involved in T-cell-priming, antibody production and cell-to-cell interactions. Therefore, it has been studied in the context of inflammatory diseases, viral infections, angiogenesis and cancer as both a prognostic biomarker and a potential therapeutic target [2].

Different associations with sSema4D levels have been previously described in infectious diseases. In patients with hemorrhagic fever with renal syndrome (HFRS) caused by the Hantaan virus, sSema4D levels were significantly higher in the acute phase compared to healthy controls [36]. Higher levels of sSema4D were associated with disease severity as wells as corresponding clinical parameters. In the convalescent phase, the sSema4D levels of patients with HFRS declined but still remained higher than in healthy controls. In patients with acute hepatitis C virus infection increased serum sSema4D levels have been observed, which dropped to the level of healthy controls after sustained virological response [37]. However, in patients with chronic HIV, sSema4D levels are decreased compared to healthy controls before and 2 years after successful highly-active anti-retroviral therapy regardless of the CD4 and CD8 cell counts [38].

In our study, we did not observe a difference in sSema4D between septic and non-septic critically ill patients. There was also no association between sSema4D levels and disease etiology, disease severity or survival. We did observe a positive correlation with leukocyte count, but not with other established markers of inflammation like CRP and PCT. Similarly, Nishide et al. found a correlation between sSema4D and neutrophil count but not with CRP in patients with AAV [19].

These findings suggest that sSema4D is not regulated in sepsis and should not be considered as a general pro-inflammatory driver in that setting. Instead, sSema4D appears to be a protein which is involved in the pathogenesis of specific diseases only. Similarly, Yoshida et al. found elevated sSema4D in the serum of patients with rheumatoid arthritis, while patients with osteoarthritis, ankylosing spondylitis and systemic lupus erythematodes had no increase in sSema4D levels compared to healthy controls [18]. Consistent with these findings, sSema4D levels in RA were independent of age and gender in our cohort.

In our study, a subgroup of patients with a history of liver cirrhosis had significantly higher levels of sSema4D. In addition, sSema4D was positively correlated with aminotransferases, as markers of hepatic inflammation, and the cholestatic liver parameters bilirubin and gamma-glutamyl transpeptidase. The majority of our patients suffered from sepsis, which is associated with hyperinflammation in the early phase. This inflammation can trigger profibrotic processes in the liver. Sema4D has recently been found to be highly expressed in human fibrotic livers and in mouse models of liver fibrosis [21]. The main source of upregulated Sema4D is activated hepatic stem cells. Knockout of Sema4D alleviated liver fibrosis in different mouse models compared to the wild-type; however, Wang et al. did not measure soluble Semaphorin 4D in patients with liver fibrosis or in mice, respectively. Nevertheless, it is reasonable to assume that increased expression of Sema4D also leads to increased shedding and elevated peripheral levels of soluble Semaphorin 4D.

We acknowledge some limitations of our study. Given its design as a single-center study, high reproducibility and technical accuracy were achieved; however, transferability of the results into other settings is limited. Our cohort was heterogeneous, including patients with various disease etiologies, and, therefore, subgroups might be underpowered to show significant changes in sSema4D levels. Moreover, we did not measure membrane-bound Semaphorin 4D on peripheral blood cells, making it difficult to understand how the up- or downregulation of sSema4D is associated with increases or decreases in shedding from, for example, leukocytes in the setting of critical illness. We also did not measure sSema4D levels at different timepoints during the course of our patients’ ICU stays, so we cannot make assumptions about changes in sSema4D levels in the later stages of critical illness and sepsis.

Another potential limitation of our study is the fact that we measured sSema4D in serum instead of plasma. We decided to do so to ensure better comparability with previous studies which had examined sSema4D serum levels in infectious diseases [38] and several autoimmune disorders [18,19,20]. Of note, sSema4D is involved in thrombus formation, during which it is shed from the cell surface of platelets. Thus, pre-analytical blood sampling procedures, which initiate coagulation processes, result in increased levels of sSema4D in serum compared to plasma levels, which further challenges interpretation [26,39].

## 5. Conclusions

Our study is the first to examine the potential of sSema4D as a biomarker in the setting of critical illness and sepsis. We found that sSema4D levels are comparable between septic and non-septic patients with critical illnesses. Soluble Semaphorin 4D had no prognostic value in our cohort. However, in a subgroup of patients with a history of liver cirrhosis, we observed significantly higher sSema4D levels. Furthermore, there was a positive correlation between sSema4D and aminotransferases, bilirubin and γGT.

## Figures and Tables

**Table 1:** Baseline patient characteristics.

Parameter	All Patients	Sepsis	Non-Sepsis	*p*
Number *n*	192	125	67	
Sex (male/female) *n*	113/79	78/47	35/32	0.226
Age (years)	64.5 (18–89)	65 (21–89)	63 (18–87)	0.663
APACHE II score	17 (2–40)	18 (3–40)	16 (2–37)	0.039 *
SOFA score	10 (0–18)	11 (3–17)	7 (0–18)	0.006 *
Charlson Comorbidity index	4 (0–16)	4 (0–16)	4 (0–13)	0.297
Mechanical ventilation *n* (%)	130 (68.0)	92 (73.6)	38 (57.5)	0.036 *
Vasopressor demand *n* (%)	115 (62.2)	84 (70.0)	31 (47.7)	0.005 *
ICU days *n*	8 (1–137)	10 (1–137)	6 (2–44)	<0.001 *
Death in ICU *n* (%)	52 (27.1)	38 (30.4)	14 (20.9)	0.214
30-day mortality *n* (%)	57 (34.5)	41 (36.3)	16 (30.8)	0.606
1-year mortality *n* (%)	88 (59.9)	65 (64.4)	23 (50.0)	0.142
sSema4D (ng/mL)	35.98 (9.99–240.00)	35.38 (9.99–240.00)	36.83 (12.26–203.30)	0.522

The median and range (in parentheses) are given unless indicated otherwise. Abbreviations: APACHE: Acute Physiology and Chronic Health Evaluation; SOFA: Sepsis-Related Organ Failure Assessment; ICU: intensive care unit. Significance of the difference between sepsis and non-sepsis patients was assessed using the Mann–Whitney U test or the Chi-squared test, respectively. *p*-values < 0.05 were considered statistically significant and are denoted by an asterisk (“*”).

**Table 2:** Disease etiology of the study population and subgroup sSema4D concentrations.

Etiology of (Non-)Sepsis Critical Illness, *n* (%)	Sepsis *n* = 125	Non-Sepsis *n* = 67	sSema4D (ng/mL)	*p*
Pulmonary	69 (55.2)		33.11 (9.99–240.00)	0.059
Abdominal	19 (15.2)		36.69 (11.12–160.27)	
Urogenital	10 (8)		34.51 (16.30–82.13)	
Other	27 (21.6)		44.62 (15.70–184.60)	
Cardiocirculatory disorder		13 (19.4)	40.38 (20.90–71.58)	0.809
Respiratory failure		10 (14.9)	34.22 (12.26–93.25)	
Advanced liver disease		13 (19.4)	41.74 (24.26–203.30)	
Other		31 (46.3)	39.12 (12.81–163.01)	

The absolute numbers and percentages of the respective subgroup (in parentheses) or the median and range (in parentheses) are given. Significance of the differences between more than two groups was assessed using the Kruskal–Wallis test. *p*-values < 0.05 were considered statistically significant.

**Table 3:** Comorbidities and their influence on sSema4D levels.

Comorbidity	sSema4D Concentration in ng/mL, Median (Range)	*p*
Diabetes (*n* = 50)	36.53 (9.99–240.00)	0.996
Liver cirrhosis (*n* = 20)	47.25 (15.14–163.01)	0.020 *
Coronary artery disease (*n* = 63)	36.69 (12.26–160.27)	0.882
Hypertension (*n* = 75)	35.38 (12.95–240.00)	0.480
Chronic alcohol abuse (*n* = 25)	32.38 (15.14–163.01)	0.144
Chronic obstructive lung disease (*n* = 25)	32.79 (12.26–160.27)	0.410
Active malignancy (*n* = 23)	31.93 (11.75–160.27)	0.188

The median and range (in parentheses) are given unless indicated otherwise. Significance of the difference between groups was assessed using the Mann–Whitney U test. *p*-values < 0.05 were considered statistically significant and are denoted by an asterisk (“*”).

**Table 4:** Correlations of clinical and laboratory parameters with sSema4D serum concentrations at ICU admission.

Parameter	r	*p*-Value
Demographics		
Age	−0.095	0.191
BMI	0.061	0.406
Markers of inflammation and blood count		
Leukocytes	0.144	0.048 *
Platelets	0.020	0.781
CRP	0.020	0.781
PCT	0.016	0.852
IL-6	−0.089	0.278
IL-10	0.143	0.155
Markers of hepatobiliary function and coagulation		
ALT	0.016	0.027 *
AST	0.258	<0.001 *
γGT	0.237	0.001 *
Bilirubin	0.170	0.019 *
Albumin	−0.013	0.888
Lipase	−0.009	0.917
INR	0.006	0.934
PTT	0.029	0.695
Electrolytes and renal function		
Sodium	−0.030	0.677
Potassium	0.103	0.157
Urea	0.028	0.701
Creatinine	0.044	0.548
Cystatin C	0.120	0.185
Net fluid balance day 1	0.001	0.986
Net fluid balance day 3	0.008	0.921
Cardiopulmonary system		
NT-pro-BNP	−0.006	0.953
Norephinephrine demand at day 1 (µg/day)	−0.084	0.257
Horovitz quotient (PaO_2_/FiO_2_)	−0.099	0.429
Ventilatory FiO_2_ demand	0.108	0.384
Metabolic markers		
Glucose	−0.072	0.324
HbA1c	−0.121	0.260
Insulin	−0.008	0.940
C-Peptide	0.091	0.395
Total cholesterol	0.076	0.347
HDL	−0.008	0.943
LDL	0.131	0.228
Triglycerides	0.201	0.012 *
Clinical scores		
Days on ICU	−0.072	0.322
SOFA score day 1	−0.040	0.727
SOFA score day 3	−0.033	0.809
APACHE-II day 1	0.022	0.791
APACHE-II day 3	−0.157	0.224

A Spearman’s rank correlation test was used to calculate the significance of correlations of a positive and negative nature. *p*-values < 0.05 were considered statistically significant and are denoted by an asterisk (“*”). Abbreviations: ICU: intensive care unit; BMI: Body mass index; CRP: C-reactive protein; PCT: Procalcitonin; IL: Interleukin; ALT: Alanine aminotransferase; AST: Aspartate aminotransferase; γGT: Gamma-glutamyl transpeptidase; INR: International normalized ratio; PTT: Partial Thromboplastin Time; NT-pro-BNP: N-terminal pro B-type natriuretic peptide; PaO_2_: Arterial oxygen partial pressure; FiO_2_: Fraction of inspired oxygen; HbA1c: Glycosylated hemoglobin A1; HDL: High-density lipoprotein; LDL: Low-density lipoprotein; SOFA: Sepsis-related Organ Failure Assessment; APACHE-II: Acute Physiology and Chronic Health Evaluation II.

## Data Availability

The data presented in this study are available on request from the corresponding author.
